# A student-participation approach to attaining sustainability on campus

**DOI:** 10.1186/2193-1801-4-S2-P8

**Published:** 2015-11-27

**Authors:** Dennis Wing-kin Wu

**Affiliations:** Faculty of Science and Technology, Technological and Higher Education Institute of Hong Kong, Hong Kong

## Background

To support the global movement for a sustainable and green environment, everyone should contribute. Higher educational institutes and their users, who are mostly students, are no exception.

A strong student-participation approach should be taken to enhance the environment and to contribute to sustainability on campus. As students are the key users of facilities on campus, their knowledge, skills and habits are key to the successful attainment of sustainable campuses. Another success factor is the selection and installation of environmentally friendly facilities, and maintenance, repairs and daily routines all targeted to saving energy, water and other resources.

In addition, using renewable resources such as solar energy for hot water, and growing vegetables and environmentally friendly plants will further contribute to attaining sustainability on campus. These provisions and activities can be made available to students who can then grow vegetables on campus for food, and grow environmentally friendly plants to enhance air quality and control the presence of mosquitoes and other unwanted insects.

A strong student-participation approach to attaining sustainability on campus will fully address the three dimensions of sustainability: economy, environment and society (see http://www.arch.hku.hk/research/BEER/sustain.htm). This paper discusses the various means and measures to foster a strong student-participation approach to sustainability on campus, such as introducing credit-earning general educational modules for students to learn about energy-saving technologies and facilities; conducting exercises to enhance skills and awareness of optimal energy and resources saving; and growing vegetables or farming on campus for food.

## Methods

The course content of selected universities relating to the student-participation approach to attaining campus sustainability is reviewed, and a new study module is proposed to enhance student participation in school management in respect of sustainability policies and projects. The aim is to foster and maintain a green culture on campus with high profile campaigns, events and activities by and for students.

## Results

To arouse awareness, interest and concern about campus sustainability, subjects related to environmental protection, natural resources conservation and sustainability are included in a core general curriculum to be studied by all students, in addition to the specially designed environmental undergraduate programme(s). Student participation and involvement in the design of credit-earning educational modules enhances recognition and commitment of sustainability on campus. Students put greater efforts into designing sustainability programmes and measures that they like and are capable of implementing on their campus.

As stakeholders in the campus community, students are invited to participate in the design and development of school sustainability policies, campus development plans, and property and facilities management strategies. In addition to policy making, students are encouraged to participate in standing committees and ad-hoc task forces to work through on-campus sustainability projects from inception to completion, including raising funds from outside private sponsors or government bodies. This is an important chance for the students to apply what they learn in the classroom to real project situations. Last but not least, students' commitment to campus sustainability is fostered and maintained by nurturing a green culture on campus through high profile campaigns, events, and activities by and for students, at all times with collaboration and sponsorship from environmental organisations. This proposed model, as depicted in Figure 1, enhances student participation in the environmental sustainability movement in general and campus sustainability in particular.

## Conclusions

The proposed new approach to attaining campus sustainability via a student-participation model for all university students will feed into students' life-long knowledge and practice for attaining sustainability not only on campus, but also in their future homes and work places, thus contributing significantly to world sustainability.

